# Innovative Approaches for Enhancing the Stability and Functionality of Essential Oils in Food Systems: A Critical and Bibliometric Review

**DOI:** 10.3390/plants15121811

**Published:** 2026-06-12

**Authors:** Neliswa H. Gcabashe, Yardjouma Silue, Olaniyi A. Fawole

**Affiliations:** 1 Postharvest and Agroprocessing Research Centre, Department of Botany and Plant Biotechnology, University of Johannesburg, Auckland Park, P.O. Box 524, Johannesburg 2006, South Africa; neliswagcabashe09@gmail.com (N.H.G.); siluey@uj.ac.za (Y.S.); 2 South African Research Chairs Initiative in Sustainable Preservation and Agroprocessing Research, Department of Botany and Plant Biotechnology, University of Johannesburg, Johannesburg 2006, South Africa

**Keywords:** essential oils, stabilization strategies, bibliometric analysis, food systems, antioxidant activity, antimicrobial activity

## Abstract

Essential oils (EOs) are widely studied as natural antimicrobial and antioxidant agents in food systems. However, their high volatility, low water solubility, instability, phytotoxicity, and strong aroma often limit their consistent applicability for food preservation. This review critically examines the literature and synthesizes current essential oil stabilization and delivery strategies in food systems, integrated with a bibliometric analysis of Scopus-indexed literature published before June 2025. The bibliometric findings showed an expanding research field, supported by 543 authors and 54 journals, revealing the disciplinary diversity of research on essential oil-based preservation systems. In addition, the review highlights a significant focus of studies on nanoemulsions, encapsulation, and active packaging in essential oil applications. Interestingly, the study also reveals the emergence of non-contact, or vapor-phase, technologies with improved release management. Furthermore, the review shows that essential oils’ functionality depends not only on major bioactive compounds but also on chemical class, oxidative sensitivity, release behavior, interactions with the food matrix, and the delivery platform. Mechanistically, stabilization technologies such as emulsions, encapsulation, and coatings/films can improve the protection, dispersion, and release of essential oils; however, their effectiveness strongly relies on formulation variables, matrix composition, and the regulatory framework. Emerging platforms such as nanofibers, zeolites, and metal–organic frameworks offer promising routes for vapor-phase or non-contact delivery systems, ensuring improved release control, functionality, and sensory quality, but may be limited by their scalability and production cost. However, a major research gap identified by this review is the imbalance between extensive “in vitro” studies and limited studies on real food matrices, which impedes understanding of the impacts of food matrices and packaging materials on essential oil release kinetics, antimicrobial efficacy, and sensory quality. Therefore, future research should integrate real-food applications, consumer acceptability, shelf-life performance, release-kinetic modeling, and techno-economic analysis to advance essential-oil-based technologies in food systems.

## 1. Introduction

The global population is predicted to reach 9.8 billion by 2050 [1], further escalating pressure on food production and preservation systems and on natural resources. Projections indicate a 50–70% increase in global food demand in the coming decades, while fresh produce losses and waste after harvest are currently estimated at 30–50% along the supply chain, resulting in food not reaching consumers [2]. These postharvest losses, driven by senescence, microbial spoilage, physiological degradation, and inadequate storage infrastructure, significantly compromise both food quality and quantity [3]. Hence, this underscores a critical need for innovative, effective, and sustainable preservation solutions to reduce postharvest losses and support global food security.

Traditionally, postharvest losses have long been mitigated using physical and chemical preservation methods, particularly synthetic fungicides, which are widely used. However, growing consumer awareness of health and environmental concerns related to chemical residues has seriously limited their adoption and use for food preservation [4]. Consequently, researchers spurred considerable interest in natural alternatives, such as plant-derived preservatives. Among these natural preservatives, essential oils have emerged as promising postharvest biocontrol agents with applications beyond single fumigation to a broader role in postharvest disease management [5].

Essential oils (EO) are complex mixtures of volatile secondary metabolites extracted from plants, commonly by processes such as steam or hydrodistillation [6]. Beyond their potent scents, EOs exhibit a wide range of activities, including antimicrobial, antioxidant, and pesticidal, due to their chemical diversity and variability. In fact, essential oils encompass a range of diverse chemical classes, including alcohols, terpenes, fatty acids, aldehydes, C6 compounds, esters, and C13 nor isoprenoids [7]. Additionally, the broad-spectrum bioactivity is associated with multiple mechanisms, including disruption of microbial membranes, alteration of cellular morphology, leakage of intracellular components, and interference with metabolic pathways [8]. In other words, EO functionality is hardly determined by a single major compound; rather, it results from interactions among major and minor constituents and their corresponding chemical classes.

However, despite their potential, the application of EOs in food systems, particularly postharvest preservation, presents certain drawbacks. Indeed, their high volatility, low water solubility, inherent chemical reactivity, and strong aroma can limit their stability, biological efficacy, and consumer acceptance. Moreover, EOs are highly sensitive to environmental stressors, such as temperature, humidity, light, and oxygen, leading to various undesirable phenomena, including oxidation, photodegradation, and loss of bioactivity [9]. Exposure to these environmental stressors may result in structural modifications of EOs’ key bioactive constituents, thereby influencing their antimicrobial and antioxidant efficacy. Moreover, the efficacy of essential oils in real food systems is strongly influenced by interactions with food components, packaging materials, and storage conditions. As a result, EO concentrations that are effective “in vitro” conditions may not necessarily produce comparable effects in real food matrices in post-harvest storage systems.

In addition to postharvest environmental conditions, preharvest factors also play a critical role in determining EO yield, chemical composition, and functional stability [10]. Abiotic stressors, including elevated temperatures, high vapor pressure deficit, salinity, and radiation, can disrupt plant physiological processes, altering secondary metabolite biosynthesis [11]. For instance, heat stress significantly affects EO production by interfering with biosynthetic precursors, thereby reducing the production of plant secondary metabolites [10]. Previous studies have reported a positive correlation between net CO_2_ assimilation, EO yield, leaf area, and trichome density per unit area [12]. This association is strongly linked to glandular trichome abundance, as these structures serve as the primary sites of EO biosynthesis.

To overcome these limitations, recent research has importantly focused on EO stabilization and on delivering strategies that enhance EO stability, control release, and preserve functionality across applications and storage [13]. Among these, nanoemulsions and microencapsulation have emerged as a valuable strategy for delivering EOs [9]. They often involve the use of diverse absorbent and carrier materials to stabilize the EOs and control their release. Moreover, active packaging, cold plasma technologies, nanofibers, porous carriers, and other advanced systems have shown promising results in further enhancing the functionality of EOs [14]. However, these technologies differ in their stabilization mechanisms, delivery behavior, scalability, safety, regulatory acceptability, and suitability for specific foot matrices or postharvest application conditions. Therefore, a critical evaluation of the existing literature is necessary to identify available technologies and understand their mechanisms, performance, specifications, and limitations. However, many existing reviews focus on a single EO, delivery system, food category, or type of carrier material (Table 1), highlighting the fragmentation of the literature on EO stabilization and delivery strategies. To clearly position the originality of the present review, Table 1 exhibits the limitations of selected existing reviews from Scopus and Web of Science and the unique contributions of this study. In addition, this review provides a global overview of the research landscape on essential oils in food systems by integrating a bibliometric analytical approach.

The study aims to comprehensively review the literature and synthesize current strategies for enhancing the stability and functionality of essential oils in food systems. Specifically, this study seeks to achieve the following:

(i) Synthesize and critically evaluate scientific evidence on both EO stabilization technologies and delivery systems for practical applications in food systems;
(ii) Map the global research landscape and emerging thematic areas in research on EO stabilization delivery strategies in food system applications;
(iii) Examine how EO chemistry, instability mechanisms, delivery platforms, and food matrix interactions influence functionality;
(iv) Highlight research gaps, technological challenges, and future directions for advancing essential oil-based solutions for food preservation.

## 2. Methodology Approach

This study was designed to provide a comprehensive, bibliometric-assisted analysis of literature on stabilization strategies and functional applications of essential oils (EOs) in food systems. Thus, to ensure transparency and reproducibility in the identification and selection of the literature, the methodological design was guided by well-established review principles, namely the Preferred Reporting Items for Systematic Reviews and Meta-Analysis (PRISMA) guidelines [25]. The study encompassed research studies addressing (i) stabilization strategies of essential oils, (ii) their bioactivity and functional properties, and (iii) their applications in food systems. Following PRISMA guidelines, data collection was carried out in four key stages: identification, screening, eligibility, and inclusion.

### 2.1. Identification

The Scopus database (https://www.scopus.com/, accessed on 30 June 2025) was used for its comprehensive coverage across food science, chemistry, agricultural sciences, biotechnology, materials sciences, and environmental sciences, all relevant to EO stabilization research. Additionally, the Scopus database provides highly accurate, rigorous indexing standards, standardized bibliographic metadata, author keywords, citation information, and affiliation data, all of which are required for reproducible bibliometric mapping. Using a single database minimizes inconsistencies that can arise when merging records from databases with different indexing structures. Nevertheless, the exclusive use of Scopus is acknowledged as a methodological limitation since other databases, such as Web of Science, can provide accurate metadata and relevant studies on EOs. Moreover, this limitation was considered in the interpretation of the bibliometric trends and in the conclusions of this review.

The search strategy consisted of defining keywords that capture studies addressing EO stabilization, stability, bioactivity, and functionality in food systems. The search string consisted of connecting keywords using Boolean operators (OR and AND), asterisks (*), and quotation marks (“…”). Hence, the specific search string was “essential oil*” AND (stabilization OR stability) AND (bioactivity* OR functionality). The search yielded 327 documents, including research articles (223), review articles (84), book chapters (14), and conference proceedings (6). These retrieved records were then refined using predefined filters and manual screening to identify studies relevant to EO stabilization and food-system applications.

### 2.2. Screening and Eligibility Criteria (Inclusion/Exclusion)

**Figure 1:**
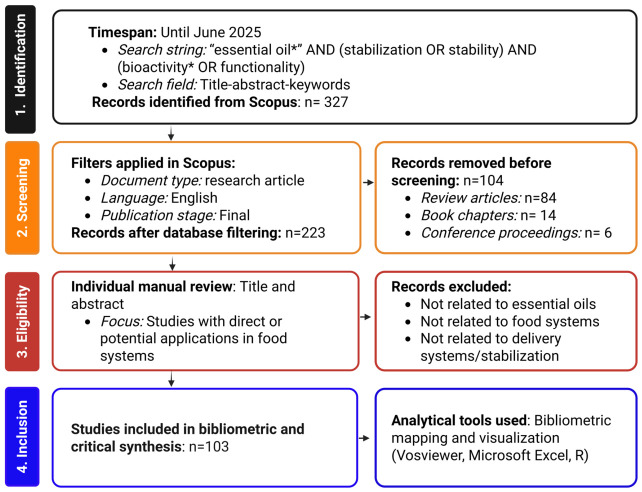
PRISMA-guided studies’ selection flow diagram for the bibliometric-assisted mapping and critical synthesis of essential oil stabilization in food systems. Asterisks (*) and quotation (“…”) marks were used to refine the database search strategy.

The screening was conducted in two phases. Firstly, the database-based filters were applied in Scopus to retain English-language research articles that reached the final publication stage. Thus, we refined our search using predefined parameters in the Scopus database, such as language (“limited to English”), document type (“limited to articles”), and publication stage (“final publication stage”). The second phase was a manual review of titles and abstracts to include only publications relevant to EO stabilization technologies, delivery systems, bioactivity, controlled release, and food system applications (food packaging, food preservation, or postharvest-related applications). In addition, both in vitro and in vivo investigations were included, while studies that focused exclusively on pharmaceutical, cosmetic, aromatherapy, or non-food-related uses of EOs, and lacked relevance to EO stabilization or delivery strategies, were excluded. As this study focuses on macro-level bibliometric mapping and thematic synthesis, a formal clinical risk-of-bias or individual experimental quality appraisal was not used as a selection criterion. Therefore, records that met all inclusion criteria were exported in comma-separated values (.csv) format for the bibliometric analysis. Figure 1 below represents a flow diagram of the data collection procedure following the PRISMA model.

## 3. Global Research Landscape of Essential Oil Stabilization in Food: A Bibliometric Perspective

### 3.1. Global Research Performance Statistics

**Figure 2:**
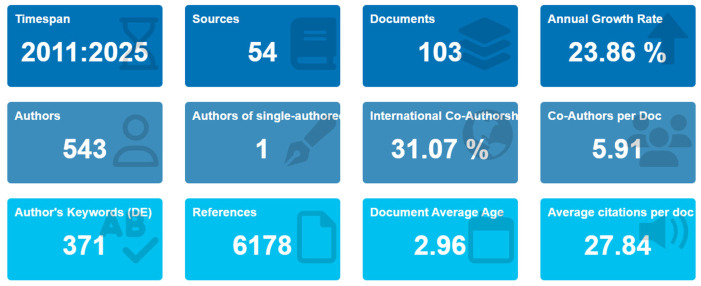
Global bibliometric performance indicators of essential oil stabilization research in food systems from Scopus between 2011 and 2025.

The timespan (2011–June 2025) reflects that research on stabilization strategies and the bioactivities of essential oils for food applications is still a relatively young yet emerging research field (Figure 2). Although the total publication volume (103 documents) remains relatively modest compared with other fields, such as edible coatings and films [25], its high-growth rate (~24%) against 16% for edible coatings and films on fruits suggests growing scientific interest in improving the stability, controlled release, and food application of EOs. This growth is likely associated with the convergence of three factors: consumer demand for natural preservatives, regulatory pressure to reduce synthetic additives, and technological needs to overcome EO limitations (oxidation, volatility, poor water solubility, and sensory intensity). Interestingly, 54 distinct journals disseminate research on stabilization technologies and bio-functional applications of essential oils, revealing the multidisciplinary nature of the field and its interdisciplinary connections across food science, biotechnology, materials science, chemistry, microbiology, and postharvest technology. This disciplinary diversity is crucial for the successful application of EOs in food systems, as it requires the smooth integration of multiple disciplines, including sensory evaluation, real-food validation, formulation science, and data science. This is supported by the diversity of authors’ keywords (371) and the high number of authors involved (543). Therefore, this bibliometric analysis, although limited by the data source, indicates the importance of an integrated review that covers chemical stability, delivery systems, and functional applications in food/postharvest systems, justifying the scope of this review.

### 3.2. Geographic Distribution and Global Collaboration Network in Essential Oil Stabilization Research

**Figure 3:**
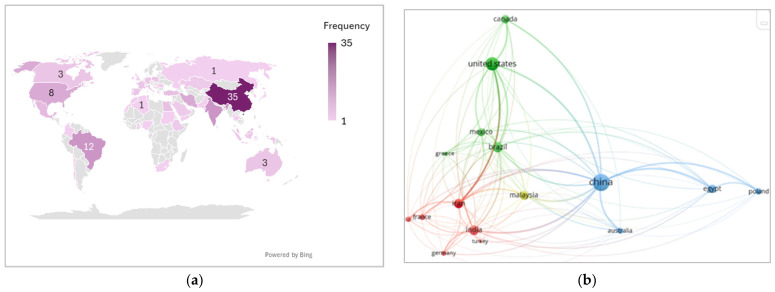
Geographic distribution (**a**) and international collaboration network (**b**) in essential oil stabilization research for food systems from Scopus between 2011 and 2025. In (**b**), nodes denote countries, node size reflects publication output, edge thickness indicates collaboration strength, and node closeness indicates the frequency of co-authorship links.

Research on essential oil stabilization techniques is an active and globally significant field, with contributions from 45 countries (Figure 3). The leading contribution of China may reflect substantial investment in bio-based materials research, food packaging innovation, and natural antimicrobial systems. Other countries, such as Brazil and India, are also major contributors to this area, likely due to their rich biodiversity, extensive agricultural sectors, and efforts to valorize plant-derived bioactives, such as essential oils, particularly for food preservation. In contrast, the limited representation of developing regions, such as Africa, does not necessarily indicate low relevance of EO-based preservation or research in the region. This may reflect structural constraints like limited access to advanced research facilities, lower research funding, and, more importantly, reduced participation in high-output bibliometric databases such as Scopus. Although the dataset is limited for generalizing the conclusion, this indicative underrepresentation is very instructive, as many African countries harbor important biodiversity and experience significant postharvest losses. These results suggest a gap between local need, biological resources, and high-quality research output. Nevertheless, regional collaboration and analytical infrastructure could help expand the global coverage of EO stabilization research. Overall, the scattering of publications across regions reflects not only scientific interest but also disparities in research ecosystems.

### 3.3. Main Research Themes Within EO Stabilization Research

**Figure 4:**
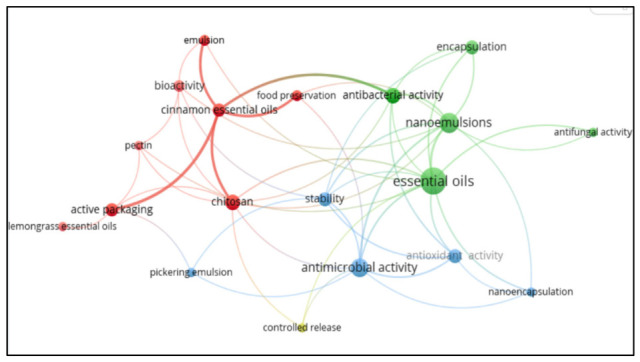
Network visualization of author keyword co-occurrence of essential oil stabilization and functional applications in food systems based on Scopus-indexed publications between 2011 and 2025. Each node represents an author keyword, with node size denoting the occurrence frequency, the link thickness designates the number of times they co-occurred, and shorter distances between nodes indicate stronger thematic relatedness. Different colors denote clusters of closely associated research themes identified from Scopus-indexed publications.

The thematic structure of research on EO stabilization and functionality in the food system was mapped through the co-occurrence analysis of authors’ keywords (Figure 4). The network visualization clearly shows that current literature on EOs in food systems is organized into three highly interconnected research clusters. This justifies the dual focus of this review on both innovative delivery strategies and bio-functionality of EOs. The first cluster (red) highlights studies on biopolymer-based systems (e.g., edible coatings and films, nanofiber mats, metal–organic frameworks, chitosan, pectin, and active packaging) with EOs as a functionalizing agent. Chitosan and pectin are the most employed biopolymers, while EOs from cinnamon and lemongrass are more widely explored for functionalizing food preservation. This cluster reflects the strong interest in incorporating EOs into polymeric matrices to potentially improve surface retention, reduce volatility, and allow gradual release.

The second cluster (green) focuses on nano-scale technologies to stabilize EOs to enhance their antifungal and antibacterial activities. This implies that improving EO dispersion, water solubility, and bioavailability persists as a dominant research direction. This cluster supports the inclusion of emulsions and encapsulation as core stabilization technologies in this review (Section 5), and cinnamon and lemongrass EOs in Table 2.

The third cluster outlines the core biological functions, particularly antimicrobial and antioxidant activities, targeted by EO delivery systems (active packaging, nanoemulsions, and Pickering emulsions). This highlights that EO stabilization research does not focus solely on protecting chemical compounds but also on preserving functional performance. However, the absence of connection with application-related keywords suggests that many studies still evaluate delivery systems and bioactivity of EOs exclusively in “in vitro” assays.

Furthermore, the isolated yet connected keyword “controlled release” signifies that it is emerging as a bridging concept between formulation technology and functional food applications. This supports the importance of discussing release kinetics, diffusion, and partitioning, and matrix interactions as central mechanisms controlling EO performance. to the three major clusters. It implies that the controlled release is an emerging property that has gained increasing interest in research on EO stabilization and their bioactivities, especially antimicrobial.

Overall, the bibliometric analysis was not conducted solely to quantify publication outputs but also to guide the critical structure of the review. Indeed, publication trends, keyword analysis, and technology-frequency patterns enabled the identification of dominant stabilization strategies, emerging delivery systems, and underexplored research areas (bottlenecks). Therefore, the bibliometric map provides a conceptual structure for the review, focusing on EO chemistry and stability mechanisms, advanced and emerging stabilization and delivery technologies, release kinetics, and food-system applications, and highlighting research gaps and future perspectives.

## 4. Chemical Characteristics and Stability of Essential Oils in Food Systems

### 4.1. Chemical Composition of Essential Oils Commonly Investigated and Their Food/Postharvest Relevance

Unlike chemical-based antimicrobial agents, essential oils are complex mixtures of chemical compounds (secondary metabolites) that may induce biological activity through synergistic or additive interactions among major and minor compounds. It is well established that variation in plant species, geographical origin, chemotype, harvest stage, extraction method, and plant part strongly affects the functional properties of EOs [26]. Therefore, Table 2 summarizes the major chemical compounds, dominant compound classes, functional properties, sources, and food system applications of selected essential oils. The most studied essential oils exhibit broader chemical classes, including phenylpropanoids, oxygenated monoterpenes, monoterpene hydrocarbons, and sesquiterpene hydrocarbons. This classification is crucial, as the EO chemical composition strongly influences volatility, solubility, oxidative stability, sensory impact, and, subsequently, EO stability, as well as its functional properties, such as antioxidant and antimicrobial activities. Indeed, monoterpene hydrocarbons are generally more oxidation-sensitive, and EOs dominated by them are more volatile [27]. Therefore, stabilization strategies, such as encapsulation, nanoemulsions, and active packaging systems (coatings and films), may be relevant for EOs dominated by monoterpene hydrocarbons before application. Additionally, controlled-release delivery systems can be particularly effective in their applications. Phenylpropanoid-rich essential oils can be highly biologically active but should be applied cautiously due to their strong aroma and potential to dominate sensory perception [27]. Consequently, understanding the chemical profile of EOs is essential for selecting stabilization and delivery strategies.

In postharvest applications, delivery systems aim to preserve the bioactivity of essential oils while minimizing phytotoxicity, off-flavor development, and volatilization. Thus, to suppress surface pathogen growth without direct contact with the commodity, vapor phase systems may be suitable for highly volatile oils. However, uncontrolled vapor release may lead to inconsistent efficacy and sensory alteration. Furthermore, edible coatings and films are more appropriate where gradual release and surface contact are required. Nanoemulsions can improve the dispersion of hydrophobic essential oil constituents and enable applications by dipping or spraying. In contrast, an encapsulated system can improve the stability of labile compounds and enable more predictable release during storage. Overall, postharvest use of essential oils should be based on a chemical-guided selection of both the essential oil and the delivery strategy.

### 4.2. Main Chemical Compounds Found in Essential Oils

**Figure 5:**
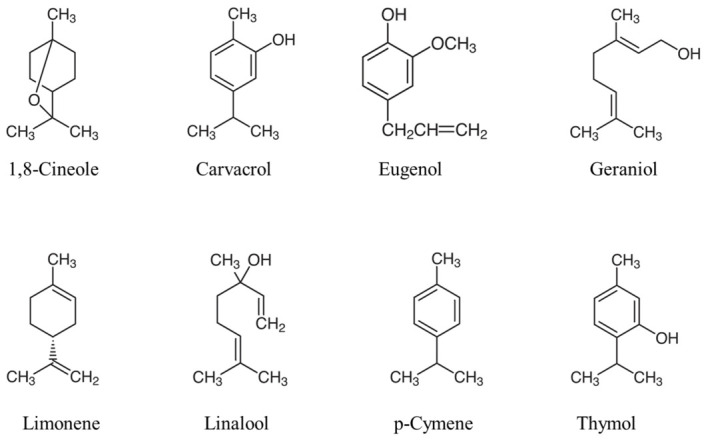
Chemical structures of major compounds commonly identified in essential oils.

The diverse chemical profiles of essential oils are mainly associated with complex mixtures of secondary metabolites, primarily monoterpenoids, phenylpropanoids, etc. As depicted in Figure 5, these compounds exhibit a wide range of structural motifs, including acyclic alcohols found in geraniol and linalool, monocyclic hydrocarbons, and aromatic phenols. These distinct chemical compounds, therefore, influence the essential oil’s antimicrobial and antioxidant properties.

**Table 2:** Chemical composition, stabilization approaches, and food system applications of the top 10 essential oils commonly used in food systems based on scientific publications retrieved from the Scopus database (2011–2025).

Essential Oils	Plant Source	Major Compounds (%)	Main Chemical Classes	Stabilization and Delivery Approaches	Main Applications in Food Systems	Chemistry-Based Interpretation	References
Cinnamon	*Cinnamomum verum*	Cinnamyldehyde (72.98%), linalool (1.80%)	Phenylpropanoids; oxygenated monoterpenes	Films, emulsions	Reduced microbial load in shrimp.	Highly bioactive with strong aroma due to phenylpropanoids; controlled release required to limit strong aroma while maintaining antimicrobial activity	[28,29,30,31,32]
Lemongrass	*Cymbopogon citratus*	Geranial (35%), nerol (29%), geraniol (8%)	Oxygenated monoterpenes	MOFs, encapsulation, films	Lemongrass EOs in starch films enhanced antimicrobial activity against *S*. *aureus* and *E*. *coli*.	Dominated by aldehydes, mainly citral, which are prone to degradation; therefore, controlled release is required to improve stability.	[33,34,35,36,37]
Thyme	*Thymus vulgaris*	Thymol (68.7%), carvacrol (34.8%)	Oxygenated monoterpene; monoterpene hydrocarbon	Film/coatings	Antimicrobial activity against *S*. *aureus*, *E*. *coli,* and *S*. *typhi*.	Sensitive to environmental stressors due to the abundance of oxygenated compounds, necessitating encapsulation for controlled release and limiting the aroma.	[38,39,40,41]
Oregano	*Origanum vulgare*	Carvacrol (77.73%), thymol (2.43%)	Oxygenated monoterpene; monoterpene hydrocarbon	Encapsulation, nanofiber mats	PCL fibers containing EOs maintained freshness and microbial growth in cherry tomatoes and blueberries.	Rich in phenolic compounds, which exhibit strong antioxidant activity via radical scavenging, supporting the need for stabilization.	[42,43,44,45,46]
Clove	*Syzygium aromaticum*	Eugenol (91%), caryophyllene (8%)	Phenylpropanoids; sesquiterpene hydrocarbons	Encapsulation, emulsions	Enhanced antimicrobial activity against Gram-positive and Gram-negative bacteria, demonstrating potential for food applications.	Abundant in phenylpropanoids (eugenol), conferring strong aroma and bioactivity, requiring stabilization.	[47,48]
Greater Galangal	*Alpinia galanga*	1,8-cineole (42.15%)	Oxygenated monoterpenes	Emulsions	EO-loaded liposomes enhanced antibacterial activity against *E*. *coli* and *S*. *aureus*.	Chemically diverse, volatility driven by oxygenated compounds.	[49,50,51]
Rose	*Rosa damascena*	Nerol (34.75%), eugenol (4.48%)	Oxygenated monoterpenes; phenylpropanoids	Emulsions	Enhanced antibacterial effects against *E. coli* and *S. aureus.*	Rich in aroma-active terpene alcohols that are highly volatile, contributing to fragrance but reducing stability.	[52,53]
Rosemary	*Rosmarinus officinalis*	1,8-cineole (9.35%), α-Pinene (20.67%)	Oxygenated monoterpenes	Films	Noteworthy antimicrobial activity against *E*. *coli* and *B*. *subtilis*, potentially for food applications.	Abundant in monoterpenes, responsible for its characteristic herbal-camphoraceous aroma. However, it is highly sensitive to oxygen, reducing stability.	[54,55,56,57]
Tea-tree	*Melaleuca alternifolia*	Terpen-4-ol (11.43%)	Oxygenated monoterpenes	Films, encapsulation	Tea tree EOs emulsions inhibited *E*. *coli* and extended strawberry shelf life by 4 days.	Mainly composed of oxygenated monoterpenes (terpinene-4-ol), responsible for antimicrobial activity but prone to oxidation, highlighting the need for stabilization.	[58,59,60]
Basil	*Ocimum basilicum*	Linalool (33.14%), eugenol (1.45%)	Oxygenated monoterpene; phenylpropanoids;	Films	Inhibited *E*. *coli* and reduced bacterial growth on chicken in cold storage.	Composed of phenylpropanoids (linalool), which drive sweet-floral aroma but are susceptible to environmental and storage-related degradation.	[39,61]

Values are presented as relative percentages reported in the cited GC-MS studies and should be interpreted as indicative ranges, as essential oil composition varies with several factors. PCL: Poly(ε-caprolactone); EOs: Essential oils; MOFs: Metal–organic frameworks.

### 4.3. Mechanisms of Instability

Essential oils, principally composed of volatile compounds, are naturally occurring substances characterized by their high vapor pressure and low water solubility. However, the inherent high volatility and chemical reactivity of EOs represent significant stability challenges during processing, storage, and applications, especially in food preservation [62]. Furthermore, various environmental factors, including exposure to light, high temperatures, air (oxygen), and humidity, can induce chemical alterations and ultimately compromise the stability and efficacy of the essential oils [62,63]. Additionally, the molecular structure of these compounds is very pivotal in their susceptibility to degradation [64].

#### 4.3.1. Autoxidation

**Figure 6:**
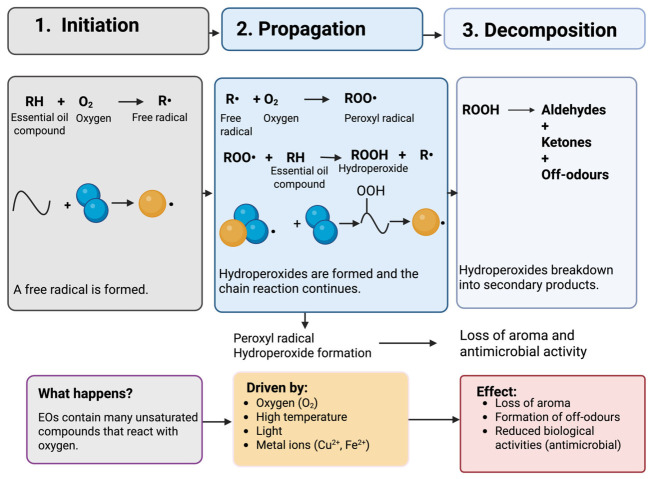
Schematic illustration of essential oil autooxidation, adapted from Turek and Stintzing [27].

Oxidation significantly influences the stability, quality, and efficacy of essential oils, leading to various chemical and sensory changes. The oxidative degradation of EOs occurs via a radical chain reaction, primarily affecting unsaturated components, which then form hydroperoxides [65]. These hydroperoxides are primary indicators of lipid peroxidation, which leads to the breakdown of EOs into various secondary products such as aldehydes and ketones [18]. Consequently, EO compounds with multiple double bonds, like monoterpene hydrocarbons, including limonene, α-terpineol, and myrcene, are highly susceptible to oxidative degradation, as unsaturated bonds accelerate radical formation and hydroperoxide generation [66]. On the other hand, phenolic compounds such as thymol, carvacrol, and eugenol can act as antioxidants by reacting with peroxyl radicals under oxidative conditions [67]. The observed antioxidant activity in EOs is directly linked to the presence of key molecular structures, such as conjugated double bonds and hydroxyl groups, which enable free radical scavenging and confer antioxidant activity. This chemical breakdown commonly leads to the development of off-flavors and rancidity, which compromise the EO’s sensory profile [68,69,70]. For instance, via oxidative transformation, oxygenated monoterpenes, such as linalool, geraniol, citral, and 1,8-cineole, may produce aldehydes, ketones, or acids that modify aroma and biological activity [66]. The rate of this degradation is driven by direct exposure to oxygen, while higher temperatures, prolonged storage time, and enzymes exacerbate this issue [32,33]. This translates directly into industrial requirements for UV-blocking, opaque active packaging films, and strict cold-chain management to control the activation energy of hydroperoxide generation. Additionally, the presence of trace elements, such as metal contaminants, acts as a strong pro-oxidant, thus reducing stability. Furthermore, oxidation involves two primary chemical mechanisms, namely autoxidation and photo-oxidation, depending on the type of oxygen involved [69]. Specifically, atmospheric triplet oxygen initiates autoxidation by triggering a damaging free-radical chain reaction that propagates along lipid hydrocarbon chains, degrading them (Figure 6).

Overall, the oxidative stability of an essential oil depends on its chemical composition, namely the presence of oxygen-containing compounds, the degree of unsaturation, functional groups (e.g., double bonds), antioxidant constituents, and storage conditions.

#### 4.3.2. Photochemical and Thermal Degradation

Photochemical degradation refers to the breakdown of volatile compounds upon exposure to light, particularly ultraviolet radiation (UV), which impairs their quality, stability, and therapeutic efficacy [70,71,72]. The degradation process can encompass both physical and chemical interactions between the EO components and singlet oxygen. This phenomenon is exemplified by the breakdown observed in compounds such as carvacrol and thymol, found in Oregano EO [72]. Furthermore, monoterpenes, a primary constituent of many essential oils, undergo rapid photodegradation, making them particularly susceptible to light [73].

Furthermore, light exposure accelerates the accumulation of oxidative compounds, leading to the loss of organoleptic properties. Hence, EO storage in the dark prevents these changes in chemical composition, underscoring light’s role in accelerating chemical reactions and altering EO stability [74,75]. A study by Turek and Stintzing [75] reported that storage conditions, particularly daylight, significantly affected the chemical composition of EOs, with monoterpenes particularly susceptible to degradation. For example, the monoterpene alpha-terpinene in rosemary oil was reduced to less than 10% of its initial concentration after only three weeks of storage at 38 °C under daylight. In contrast, its concentration remained unchanged over the same period stored at 25 °C in the dark [76]. In addition, correlating peroxide values with conductivity and pH during storage was proposed as a valuable approach for comprehensively assessing an EO’s storage history and overall quality.

Temperature plays a critical role in determining the stability of essential oils. In general, chemical reaction rates increase with temperature [27]. As a result, processes such as autoxidation and hydroperoxide decomposition are accelerated by heat, promoting the initial formation of free radicals [77]. These processes lead to the decline of terpene hydrocarbons, e.g., myrcene, and an increase in products such as p-cymene and polymerization products [66]. Therefore, across commercial supply chains, essential oil systems are exposed to environmental stressors that can significantly compromise their stability and functional performance. Fluctuations in temperature during storage and transportation accelerate oxidative degradation of sensitive EO components, reducing both antimicrobial and antioxidant properties.

#### 4.3.3. Enzymes

Enzymes play a direct role in the deterioration of EOs. Specifically, enzymes like cytochrome P450 and glutathione S-transferase can catalyze the oxidation of EOs, generating reactive oxygen species (ROS) that ultimately induce EO degradation and an altered chemical profile [78]. The activity of enzymes such as polyphenol oxidase can significantly reduce EOs’ antioxidant capacity, thereby increasing their susceptibility to oxidative degradation. However, enzymes are frequently used to enhance EO extraction; for example, cellulase and hemicellulase are used to break down plant cell walls, thereby improving yields. Conversely, this enzymatic processing can alter the EO’s chemical composition, as demonstrated by studies [79] showing that enzymatic pretreatment can simultaneously increase yield and alter the oil’s composition, thereby affecting its stability. For example, using cellulase and hemicellulose on *Rosmarinus officinalis* led to a decrease in the key component 1,8-cineole, thereby impacting the EO’s properties and stability [78,80].

### 4.4. Interaction with Food Matrix

The migration process from food contact materials into the food matrix significantly impacts food quality, safety, and flavor [81]. This phenomenon refers to the transfer of chemical contaminants, including low-molecular-weight substances like additives and oligomers, from packaging films into food products, primarily via diffusion [82]. Likewise, the interaction between essential oils and food matrices is a major determinant of their preservative efficacy, sensory acceptability, and release behavior. Thus, despite potential benefits, the industrial application of essential oils remains limited due to the inherent risk of these compounds migrating into the food product, which can lead to unintended alterations in sensory characteristics [81]. Current studies on meat, fruit, and dairy products have consistently demonstrated a concentration-dependent relationship, where higher EO levels improve microbial inhibition but simultaneously reduce organoleptic acceptability [83]. Indeed, once EO is incorporated into a food system or packaging material, its constituents may unevenly diffuse and partition between lipid, aqueous, and gaseous phases in accordance with their volatility, polarity, hydrophobicity, and affinity for food components. Therefore, this significantly affects the concentration of available active compounds and, thereby, affects microbial control and flavor perception [84]. For instance, food constituents, such as proteins, lipids, and other compounds within the food matrix, can bind, entrap, or adsorb essential oil constituents via hydrophobic interactions, hydrogen bonding, and Van der Waals forces, thereby altering the antimicrobial activity of EOs [85]. Furthermore, matrix microstructure, viscosity, water activity, and lipid distribution may influence EO diffusion kinetics and mass transfer behavior, thereby affecting the release rate, spatial distribution, and bioavailability of active compounds within food systems. This matrix-dependent interaction may explain the frequent trade-off observed between the high antimicrobial activity of EOs in an in vitro assay and their activity in actual food matrices. For example, the strong binding between eugenol and fat globules in milk necessitates the use of higher eugenol concentrations to achieve microbial inhibition in milk-based products [86,87]. In sum, the food matrix composition importantly reduces the bioavailability and effective concentration of the EO’s active components, hindering their desired application [88].

This challenge underscores the need for improved strategies to minimize premature release while retaining the bioactive potential of essential oils throughout the product’s shelf life. However, there is a critical need to balance functional efficacy with consumer acceptability, particularly regarding flavor and aroma, which are often altered during migration. Consequently, stabilization and delivery strategies should not only protect EOs from degradation but also regulate their diffusion, partitioning, and release within specific matrices.

## 5. Advanced Technologies for Enhanced Stability and Controlled Release

Essential oil stabilization strategies aim to protect volatile compounds, improve dispersion, regulate release, maintain biological activities, and reduce sensory impact, while remaining industrially applicable and regulatory acceptable. Hence, the compatibility of each technology or method depends on several factors, including the EO’s chemical profile, the target food matrix, hydrophobicity, molecular interactions, the desired release mechanism, and storage conditions. These properties largely determine the suitability of specific polymers or carrier systems for EO encapsulation and stabilization. Consequently, the selection of the delivery system is based on its ability to improve encapsulation efficiency, reduce oxidation, and enhance dispersion in aqueous systems. Therefore, the following sections discuss the mechanisms, scalability, practical applicability, and potential challenges of various stabilization strategies.

### 5.1. Emulsions/Nanoemulsions

Emulsions are essential structures in food systems, serving as dispersions of two immiscible liquids (e.g., oil and water) that are stabilized by emulsifiers. Their primary functional roles include the delivery and protection of diverse food ingredients [89,90]. Emulsions employed in food systems comprise a diverse range of structures, categorized by the dispersion medium and droplet size. Specifically, the most fundamental categories include oil-in-water (O/W) emulsions, in which oil droplets are dispersed throughout an aqueous phase, and water-in-oil (W/O) emulsions, in which water droplets are dispersed in a continuous oil phase [91]. Moreover, specialized systems like microemulsions and nanoemulsions are utilized, which are distinguished by the droplet sizes (20 to 200 nm for nanoemulsions). The nanoscale of the nanoemulsions confers them enhanced stability and optical clarity, making them ideal carriers for bioactive compound delivery [92]. Nanoemulsions and microemulsions are typically fabricated using molecular stabilizers such as surfactants or polymers. The category extends to Pickering emulsions, which are stabilized by solid colloidal particles rather than traditional surfactants, offering unique stability mechanisms. Lastly, high internal phase emulsions (HIPEs) exhibit a very high dispersed-phase volume fraction, an important characteristic for the development of structured food matrices. Therefore, emulsions are extensively utilized as vehicles for the efficient stabilization and controlled delivery of bioactive compounds [93]. Among these bioactive compounds are essential oils, which pose inherent application challenges due to their hydrophobic nature [94].

The emulsion method ensures that EOs exhibit enhanced functional performance by preserving their bioactive compounds. The clustering map (Figure 4) further provides a clear visual representation of the relationships among different stabilization strategies reported in the literature, with emulsification-based strategies emerging as a central cluster. In these systems, EOs are encapsulated as dispersed droplets within a continuous aqueous phase, thereby facilitating their delivery into hydrophilic matrices [16,95]. Crucially, the hydrophobic core of O/W nanoemulsions physically shields EOs from direct exposure to the external environment, thereby enhancing their stability and bioavailability. Nanoemulsion and Pickering emulsion systems have been investigated as delivery strategies for Cedarwood essential oil (CEO), with distinct differences in their physicochemical and stability characteristics. Furthermore, nanoemulsions commonly formulated with higher surfactant concentrations are generally associated with smaller droplet sizes (135 nm) and higher zeta potential. On the other hand, Pickering emulsions stabilized with lower amounts of solid particles, such as starch (~1%), generally form larger particles (~626 nm) with lower zeta potential. Despite these significant differences, encapsulation approaches have been shown to enhance the functional properties of CEO, including antioxidant and antibacterial activities, compared with non-encapsulated oil [96].

From an application perspective, emulsion and nanoemulsion systems are among the most practical approaches for improving EO dispersion in aqueous food environments, such as coatings and dipping treatments. Their principal advantage is increased interfacial contact between hydrophobic EO compounds and microbial cells, thereby enhancing antimicrobial activity at lower doses. However, this activity is highly associated with droplet size, emulsifier type, interfacial stability, pH, and storage temperature [97,98,99]. Furthermore, conventional emulsions can lead to creaming, coalescence, and phase separation, whereas nanoemulsions require higher energy input, which may increase production costs and raise regulatory concerns. Therefore, although emulsion-based systems are relatively scalable and technologically accessible, their industrial adoption requires careful formulation adjustments to optimize stability, sensory impact, and compatibility with the food matrix.

### 5.2. Encapsulation

#### 5.2.1. Microencapsulation

Numerous technologies are widely employed to address stability issues and improve the delivery of essential oils. The main purpose of microencapsulation is to enhance the solubility and bioavailability of essential oils by promoting controlled or gradual release and eliminating interaction with the food matrix [100]. Microencapsulation is an innovative technology in which substances, such as volatile liquids, are enclosed within a polymeric material to form microscopic capsules [101]. The diameter of microencapsulated materials ranges between 100 and 500 µm [102]. Essentially, a microcapsule consists of a core and a shell; the core contains the active ingredient (essential oils), and the surrounding polymer forms the shell, which isolates the core from the external environment [101].

In encapsulation systems, wall materials are utilized to surround and protect bioactive compounds, forming a physical barrier that isolates the core material from adverse environmental conditions [103]. The wall material used for encapsulation should have excellent film-forming and barrier properties against factors such as temperature, light, and oxygen [7]. Typically, wall materials used are insoluble and non-reactive with the core material, which is the active ingredient [101]. Therefore, wall materials can be made from gums, proteins, synthetic polymers, lipids, and, most importantly, polysaccharides. Microencapsulation techniques are classified into physical, chemical, and physicochemical methods. Encapsulation methods can broadly be categorized by their operational principles. Physical techniques, such as spray drying, freeze-drying, emulsification, and extrusion, are notably temperature-dependent [104]. In contrast, chemical and physicochemical techniques offer an advantage because they are not reliant on environmental factors such as temperature [105]. Specifically, chemical approaches include in situ polymerization. Meanwhile, physicochemical techniques include coacervation, ionic gelation, and liposomes [106].

#### 5.2.2. Nanoencapsulation

Nanoencapsulation offers significant potential to enhance the effectiveness of essential oils in food systems. In this context, nanoencapsulation or nanosystems protect against essential oils from degradation, which is often influenced by various environmental factors, thereby masking the EOs’ aroma and avoiding negative interactions with the food matrix, thereby enhancing bioactivity and targeted release. Currently, various agents are utilized to facilitate the release of EOs, including starch, guar gum, gum arabic, chitosan, cellulose, and cyclodextrin [107].

Several nanoencapsulation techniques are commonly employed, including spray drying, coacervation, liposome formation, nanoemulsion, and freeze-drying. Among these, liposome-based systems have been explored for their ability to enhance stability and bioactivity. For instance, Risaliti et al. [106] investigated nanovesicles loaded with *Salvia triloba* and *Rosmarinus officinalis* essential oils and reported stable liposome-based formulations that remained stable for over one month, with notable bioactivities, including antioxidant, anti-inflammatory, and antibacterial properties. Recent studies have highlighted the potential of biopolymer-based systems for encapsulating plant-derived compounds. For instance, guar gum/Ag-Cu nanocomposite films have demonstrated antimicrobial activity against pathogens such as *Listeria monocytogenes* and *Salmonella enterica sv. typhirium* [108]. Among encapsulation strategies, coacervation has emerged as an effective technique for protecting and delivering plant-derived products in the food industry [109]. This method relies on electrostatic interactions between oppositely charged molecules, enabling the formation of a coating matrix that efficiently entraps bioactive compounds [110]. Furthermore, in complex coacervation, essential oils and other bioactives are incorporated into a network formed by interactions between positively charged biopolymers, such as chitosan, and negatively charged counterparts, e.g., gelatin [95]. This versatility enables the technique to encapsulate both polar and nonpolar molecules, thereby enhancing its applicability in food systems.

Overall, encapsulation (nano- or micro-) is particularly important for EOs rich in highly volatile or oxidation-sensitive compounds. It can reduce evaporation, limit oxygen exposure, and enable gradual release during storage. Encapsulated systems are reported to be more effective for long-term stability and protection than simple emulsions [111]. However, carrier materials and processing methods determine the encapsulation efficiency, loading capacity, release behavior, and industrial applicability. For instance, spray drying can be relatively scalable and cost-effective, but heat exposure may reduce the retention of thermolabile EO constituents. Moreover, complex coacervation, liposomes, and polymeric nanoparticles can improve protection and release control, but their high costs, more complex processing, and regulatory uncertainty limit their scalability.

### 5.3. Active Packaging

Packaging plays an increasingly vital role in the food industry’s storage and transportation. Consequently, its primary responsibilities are to maintain physical integrity, hygienic conditions, safety, and the overall quality of the commodity [112]. Moreover, packaging should protect against environmental factors, including oxygen, humidity, and contaminants (fungi, bacteria, and insects) [113]. Thus, packaging is a promising technology that is often functionalized with bioactive agents to reduce microbial spoilage while preserving the quality [112].

Active packaging can be categorized into chemoactive and bioactive types. They are designed to extend the shelf life or improve the conditions of packaged food. Hence, they deliberately incorporate components that release or absorb substances from either the food or its surrounding environment [114]. For instance, when active agents are encapsulated within the packaging material, they release active compounds that improve the quality and safety of the food products. Among natural bioactive agents, essential oils have gained popularity due to their antimicrobial and antioxidant activities. However, numerous beneficial properties of natural bioactive agents, including EOs, are limited by their low stability, which is influenced by environmental factors such as temperature, oxidative agents, and UV radiation [82].

Essential oils are incorporated into packaging matrices (films and coatings), forming active packaging [81]. In this context, coatings are defined as films applied directly on the surface of an edible product. Upon incorporation into the food package, essential-oil-derived compounds are released in a controlled manner, enhancing the food’s properties [115]. Various materials are employed to develop edible coatings, leading to four types: polysaccharide-based, lipid-based, protein-based, and composite-based [25]. Polysaccharide-based coatings, such as starch, chitosan, pectin, and mucilage, are hydrophilic and primarily function as moisture barriers. Among these, chitosan has attracted considerable attention due to its film-forming and antimicrobial properties. For instance, Ojagh et al. [116] investigated the incorporation of cinnamon essential oils into chitosan coatings and reported enhanced antimicrobial efficacy against spoilage microorganisms in rainbow trout fillets. The coated fillets maintained sensory quality for up to 16 days, extending shelf life and preserving quality. Conversely, protein-based coatings, exemplified by zein and whey protein, are commonly used for their excellent gas barrier properties and for safeguarding against oxygen and moisture [117]. For instance, a study by Moradi et al. [118] demonstrated that a coating made from corn protein combined with *Zataria multiflora* and *Boiss* essential oils effectively inhibited the growth of *Listeria monocytogenes* and *E. Coli* in beef. Alternatively, composite-based coatings combine water-soluble colloids with lipids. A coating containing glycerol, sodium alginate, and lemongrass essential oil successfully inhibited microbial growth and maintained the physicochemical properties of fresh-cut pineapples [119].

Active packaging is particularly relevant for postharvest applications, as it provides surface retention and the gradual release of EO constituents at the fruit or food interface, making them suitable for controlling surface microbial growth. However, active packaging requires a perfect balance between antimicrobial activity, barrier properties, mechanical integrity, migration behavior, sensory quality, and compliance with food-contact regulations. These restrain their scalability and industrial adoption.

## 6. Emerging Delivery Systems for Controlled Release of Essential Oils

### 6.1. Nanofiber Mats as Platforms for EO Stabilization

Due to their high surface-to-volume ratio and exceptional porosity, nanofiber mats have emerged as a critical material in various fields, including tissue engineering, wound dressing, drug delivery, and energy storage [120]. These mats are typically fabricated using various spinning techniques. It involves two main groups of techniques: electrospinning, which uses an electrostatic force to create fibers, and non-electrospinning methods such as phase separation, which rely on mechanical forces [121]. Furthermore, different nanofiber structures can be achieved using these fabrication methods, including core–shell, bicomponent, hollow, and porous [122]. For instance, coaxial electrospinning is used to produce core–shell structures, while hollow nanofibers are fabricated via methods such as chemical vapor deposition. Similarly, porous nanofibers are commonly fabricated using phase separation [112].

Electrospinning is a promising method for effectively incorporating a wide range of active agents, such as antioxidants and antimicrobials, into nanofiber mats [123]. Research has demonstrated that the favorable nanostructure of electrospun fibers, characterized by a high surface area-to-volume ratio, is highly effective for controlled delivery applications. This morphology enables the sustained release of encapsulated bioactive compounds, amplifying their efficacy at the food surface to a significant degree [124]. Consequently, the electrospinning technique offers a promising method for nano-level entrapment of essential oils, thereby enhancing their stability and utilization. Electrospun nanofibers often exhibit specific properties, such as biodegradability, mechanical strength, and bioactivity, which are directly influenced by the choice of base material and the type of incorporated essential oil. Additionally, combining materials such as polymers offers promising opportunities for applications in active packaging and controlled-release systems [120]. Zein has been extensively explored as a protein-based biopolymer for the fabrication of nanofiber mats intended for food systems. These nanofibrous matrices have been successfully employed to encapsulate essential oils such as *Foeniculum vulgare* and *Carum carvi*, highlighting their suitability as carrier systems for volatile bioactive compounds [125]. Owing to their favorable functional characteristics, including effective oxygen and light barrier properties and high thermal stability, zein-based nanofiber mats improve preservation performance.

Overall, nanofiber-based systems are promising for active pads, liners, and non-contact packaging systems. However, the current body of research remains limited and largely confined to laboratory-scale fabrication, with a lower technological readiness than emulsions, edible coatings, and packaging films. Additionally, these nanofibers face key barriers, including solvent residues, fiber uniformity, mechanical strength, regulatory approval, and consumer acceptance of food in contact with nanomaterials. Consequently, further studies are needed to expand their application across a wide range of green preservation systems and to better establish their long-term stability and industrial scalability in food systems.

### 6.2. Zeolites

Zeolites are a class of tectosilicates, naturally occurring or synthetically produced. They are characterized by an open lattice framework composed of silica and alumina tetrahedra [126]. This unique structure yields molecular-sized pores of varied dimensions and geometries, making them highly effective for a range of sustainable processes. Furthermore, zeolites have been adapted to create antimicrobial systems, typically by incorporating metal ions such as Silver (Ag), Copper (Cu), or Zinc (Zn). The growing interest in zeolites is attributed to their high surface area, large pore volume, surface versatility, biocompatibility, and economic synthesis [127]. When integrated into antimicrobial complexes, zeolites serve a dual role; they function effectively as carriers for active agents while providing intrinsic inorganic antimicrobial properties. The inherent pore structure of zeolites is crucial to their performance, as it not only facilitates bacterial adhesion but also enables the controlled release of EOs, thereby enhancing antimicrobial efficacy. Furthermore, EO stabilization occurs through physical adsorption; this process is driven by the Van der Waals interactions and electrostatic attraction occurring on the zeolite’s polar surface [128]. The antibacterial efficacy of thymol and carvacrol has been further enhanced through encapsulation using supercritical solvent impregnation into natural clinoptilolite zeolite [127]. These composite systems consistently demonstrate broad-spectrum antibacterial activity against both Gram-negative and Gram-positive bacteria, including *Escherichia coli* and *Staphylococcus aureus* [129]. Across literature, *E*. *coli* is generally more susceptible to these composites, exhibiting inhibition at lower concentrations than *S*. *aureus*, which aligns with established differences in the structural and permeability characteristics of their cell walls [127]. Zeolites are suitable for vapor-phase or non-contact antimicrobial packaging due to their thermal stability and their porous structures for controlled release. However, despite their promising potential, zeolites still lack a robust toxicological framework, regulatory oversight, and robust food validation studies.

### 6.3. Metal–Organic Frameworks

Metal–organic frameworks (MOFs) represent a relatively new class of crystalline porous materials constructed from metal ions or clusters coordinated to organic ligands [130]. MOFs are particularly well-suited for EO encapsulation given that they possess a much larger surface area, ranging from 1000 to 10,000 m^2^/g and larger pore sizes [131]. Therefore, they represent a cutting-edge material for immobilizing volatile compounds by encapsulating EOs within their porous structure, thereby facilitating controlled release. This not only prevents the early evaporation of EOs but also optimizes their antioxidant and antimicrobial potential, significantly enhancing the preservation capabilities of active packaging [132]. The Zeolite Imidazole Framework, constructed from Zn^2+^ nodes coordinated with a 2-methylimidazole ligand, is one of the most widely investigated MOFs. ZIF-8’s popularity stems from its easy synthesis, high stability, and specialized adsorption properties [133].

Wu et al. [132] were among the first studies demonstrating the use of a metal–organic framework as a carrier for volatile antimicrobial EOs. In their study, a Zinc-based MOF was specifically synthesized and successfully loaded with thymol at 3.96%. The thymol-loaded ZnMOF exhibited potent antibacterial activity against a three-strain cocktail of *E. coli* 0157:H7. This sustained antimicrobial activity was attributed to the controlled release of thymol, achieved by its incorporation into the porous MOF structure. This work highlighted an effective antimicrobial agent with promising potential for indirect applications in enhancing food safety.

Despite the strong potential of MOFs as smart carriers for bioactive compounds, their application in food systems remains at an early stage. In aqueous solutions, Cu^2+^ and Zn^2+^ based MOFs are preferred due to their intrinsic antimicrobial activity and low-cost synthesis; however, their poor hydrolytic stability often leads to framework degradation and uncontrolled release of encapsulated EOs, thereby reducing performance [134].

### 6.4. Absorbent Pads

Fresh food products, such as meat, poultry, and minimally processed fruits and vegetables, naturally release exudate during storage. This liquid poses a significant issue because it can negatively affect the sensory qualities of packaged food and accelerate microbial spoilage [135]. Furthermore, the exuded liquid is a rich growth medium for pathogens and spoilage microorganisms due to its high nutrient concentration, leached from the commodity [136]. Consequently, the food industry has long relied on absorbent pads to manage this risk and protect consumer acceptance [137]. These pads are strategically placed in the packaging, for instance, at the base of trays to capture the liquid exudate. Products utilizing this packaging method commonly include fresh meat. A key area of recent progress in food packaging involves the novel use of EO-loaded absorbent pads to improve safety and preservation [138].

Liu et al. [135] reported that incorporating essential oils has emerged as an effective strategy for extending the shelf life of refrigerated poultry products. Essential oil-loaded pads have consistently been shown to suppress microbial growth and limit lipid peroxidation and protein oxidation, thereby preserving sensory quality during cold storage. Formulations containing *Carum copticum* and chamomile EOs demonstrate pronounced antimicrobial and antioxidant effects against both spoilage microorganisms and foodborne pathogens, including *Staphylococcus aureus.* Collectively, these findings highlight the potential of essential oil-based active absorbent pads as functional packaging systems capable of enhancing the microbial safety and oxidative stability of poultry products during refrigerated storage.

## 7. Overview of Materials and Fabrication Technologies for Enhanced Stability and Controlled Release

**Figure 7:**
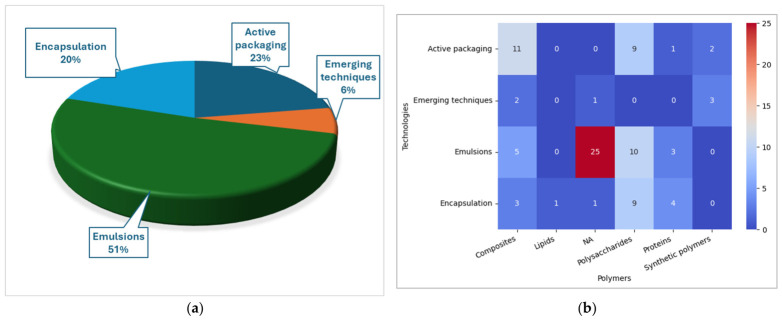
Distribution of fabrication technologies used in EO delivery systems (**a**) extracted from the Scopus database, (**b**) The heat map further quantifies the number of studies linking fabrication technologies with classes of polymer materials.

Based on the 103 documents retrieved from the Scopus database, Figure 7a illustrates that emulsions are the most widely researched and applied delivery system, accounting for 51% of the reported studies. This distribution further elaborates on the conceptual discussion of advanced technologies to enhance the stability of essential oils. Particularly, emulsions dominate literature due to their extensive exploration and represent the largest proportion, which might be driven by their technological accessibility and relatively scalable potential. Encapsulation and active packaging constitute the mid-range categories, reflecting their growing relevance in enhancing stability, protection, and targeted release of essential oils, as well as improving product shelf life through functional packaging systems. In contrast, emerging techniques represent only 6% of the retrieved literature. This indicates that although novel and advanced approaches are being explored, research efforts remain concentrated on well-established strategies. Nevertheless, there is a noticeable shift toward strategies that promote non-contact or vapor-phase applications, particularly systems that enable controlled release of EOs. Despite the utility of nanoemulsions in food systems, several critical limitations have driven increasing research interest in alternative delivery systems such as nanofiber mats, zeolites, and metal–organic frameworks (MOFs). These challenges include the need for high concentrations of surfactants and oils in nanoemulsion formulations, which may induce cytotoxicity and pose safety concerns, especially in food applications [139]. Furthermore, while active packaging is a promising strategy for enhancing food quality, its practical implementation is constrained by inherent factors, such as coating instability over time, material compatibility across diverse food matrices, and sensory alterations [140].

Figure 7b highlights the prevalence of various material classes, including proteins, polysaccharides, synthetic polymers, and lipids. Biopolymer-based carriers are among the most attractive materials for EO stabilization and application in food systems. This may be due to their biodegradability, wide availability, and compliance with food safety standards. The formulation of emulsions is mostly stabilized by surfactants, denoted as NA (not available), followed by polysaccharides. The preference for polysaccharides stems from their exceptional ability to form thick, strong interfacial membranes at the oil-water interface via steric hindrance and electrostatic interactions [141]. On the other hand, composite materials are particularly preferred because individual biopolymers exhibit distinct properties, such as moisture and oxygen barriers, mechanical strength, and performance under specific environmental conditions. Therefore, biopolymer-based systems require blending, crosslinking, or reinforcement to achieve industrially relevant stability and functionality. Our bibliometric findings reveal that edible coatings/films are most often formulated with composite materials (Figure 7b), generally composed of polysaccharides and proteins. Polysaccharides are known for their film-forming capacity and EO-loading capacity, while proteins may offer emulsifying and interfacial stabilization properties [142]. The blending approach enhances the coating’s overall effectiveness, particularly its mechanical strength and barrier properties. Although electropsun nanofibers offer high surface area and tunable release, their industrial adoption remains limited by production scale, cost, and the synthetic nature of the materials. Synthetic polymers can provide stronger mechanical stability, lower degradation, and more predictable release than many natural polymers, making them relevant to electrospun fiber systems. However, their use in the food system must consider biodegradability, migration limits, consumer perception, and regulatory approval.

## 8. Release Modeling and Formulation Optimization for Controlled EO Delivery

Controlled release is a pivotal determinant for a successful application of essential oils in food systems. Indeed, EO efficacy does not rely solely on the total amount loaded into the carrier material, but also on the location, rate, and duration of compound release. A sharp release may deliver high antimicrobial activity but can lead to phytotoxicity (physiological disorders), sensory rejection, or premature loss of the active compound. Conversely, slow release may fail to achieve the required antimicrobial activity.

Therefore, release modeling, a quantitative tool used to describe the kinetics and mechanisms of compound release, becomes very useful for understanding release kinetics. Additionally, optimization is crucial for maintaining bioactivity and ensuring controlled release over time. Release from encapsulation matrices is commonly described using established kinetic models, including zero-order, first-order, Higuchi, and Korsmeyer-Peppas models. These models account for concentration-dependent release, diffusion-controlled transport, and mechanistic diffusion-relaxation behavior within polymeric systems [83]. Furthermore, formulation optimization provides insight into identifying the optimal combination of carrier materials, concentrations, and process parameters to achieve consistent, sustained release profiles. Therefore, multivariate optimization methods have attracted attention for improving analytical processes and the conditions under which bioactive compounds or substances are encapsulated. Various designs have been employed, including the Central Composite Rotatable design (CCRD), Central Composite design (CCD), Box–Behnken design (BBD), and D-optimal design (DSD) [143]. Hamid et al. [144] employed a D-optimal design to optimize the extraction and microencapsulation of Basil essential oils, aiming to enhance both the yield and encapsulation efficiency. The study identified optimal conditions: 0.58% pectin, 0.41% casein, pH 3.4, and stirring at 1900 rpm for 16.8 min. This resulted in a predicted microencapsulation yield of 76% and efficiency of 92.8%. Experimental validation closely aligned with these predictions, with an observed yield of 80.45%, demonstrates the effectiveness of statistical optimization in guiding encapsulation processes.

Recently, Machine Learning (ML) has been integrated as a complementary tool for optimizing complex systems, such as nanoemulsion formulations and drug/food delivery systems [145]. Artificial intelligence enables researchers to efficiently develop hypotheses, analyze datasets, and derive insightful conclusions that significantly reduce the time and effort required by conventional methods [83,145]. Algorithms such as Artificial Neural Networks (ANNs) and Genetic Algorithms (GAs) have been employed to accurately predict parameters, including droplet size and stability, as well as the encapsulation efficiency of EOs [146]. In addition, tools like Support Vector Machines (SVMs) are used for robust classification, such as sorting emulsion stability. The successful validation of these models, where experimental results closely match highly accurate predictions, confirms the robustness and reliability of AI/ML and holds great promise for developing more efficient, cost-effective, and precise nanoemulsions for the food industry [147].

Release modeling and formulation optimization shift EO stabilization research from empirical trial-and-error toward rational design using ML tools. By linking formulation variables to release kinetics, stability, antimicrobial activity, and sensory perception, these approaches help identify delivery systems that are reliable in real food and postharvest environments.

## 9. Applications in Food Systems: Balancing Stability, Release, and Quality

Research has consistently shown that essential oils (EOs) have significant potential for postharvest preservation by effectively mitigating microbial spoilage and maintaining the physicochemical attributes of fresh produce, such as meat, fruits, and vegetables. Numerous EOs have been extensively documented for their potent in vitro and in vivo activities [148]. So, this potential directly translates into reduced microbial spoilage across a wide range of produce, including grapes, strawberries, and okra. Moreover, EOs enhance the antioxidant capacity while preserving the nutritional quality of the produce [86,149].

In food systems, EOs have been demonstrated to effectively prolong the shelf life and reduce postharvest losses. For instance, Sobhy et al. [150] reported that thyme, clove oil, neem, and clove essential oils positively influenced the postharvest preservation of ‘Amrapali’ mangoes. Likewise, the thyme EO (0.1%) yielded the best overall fruit quality, whilst extending shelf life by three days and controlling the decay by approximately 90% compared to untreated fruit. Furthermore, the efficacy of EOs in food preservation goes beyond fruits and vegetables. Indeed, EOs have demonstrated a broad-spectrum of antimicrobial activity against major foodborne pathogens, including *Escherichia coli, Listeria monocytogenes,* and *Salmonella typhimurium*. Vapor-phase application has emerged as an effective strategy, enhancing antimicrobial activity while minimizing direct contact with food matrices. For instance, thyme EO vapors significantly reduced microbial load in dried meat when applied as vapors [148]. Additionally, [151] explored Cardamon EO, focusing on 1,8-cineole as a major constituent, demonstrated significant antibacterial activity against key foodborne pathogens, including *Listeria monocytogenes*, *Staphylococcus aureus*, *Escherichia coli*, and *Salmonella typhimurium*. Mechanistically, the antimicrobial effect is primarily due to 1,8-cineole-induced disruption of the bacterial cell membrane, leading to leakage of intracellular components. These findings highlighted the potential of Cardamom EO as a natural preservative for improving the microbial safety of perishable foods.

**Figure 8:**
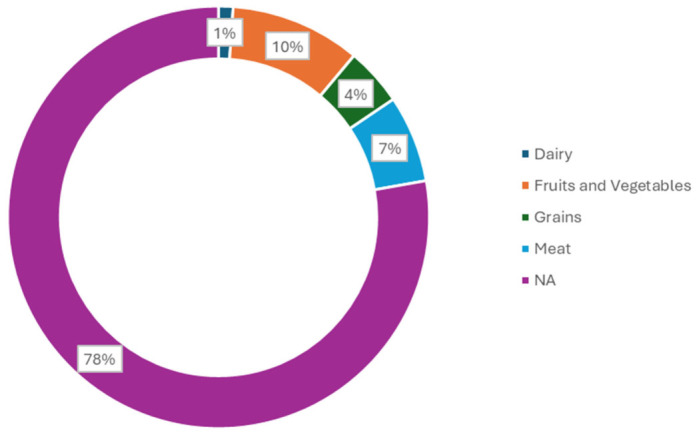
Distribution of studies on essential oil applications in food systems. NA: Not applied.

The present review highlights that many studies (78% of documents retrieved from the Scopus database) on the use of essential oils in food systems are conducted under in vitro conditions, while the application in real food matrices remains limited (Figure 8). Among food-based application studies, fruits and vegetables are the most frequently investigated (10%), followed by meat (7%) and grains (4%). The high proportion of in vitro studies highlights a critical translational gap in the current literature, underscoring the need for future research to evaluate the antimicrobial and antioxidant performance of stabilized EOs in complex food matrices. This aligns with the directions suggested by Mani-López et al. [152], who indicated that future work should focus on understanding EO-food matrix interactions, assessing long-term stability and release behavior under realistic storage conditions, and ensuring safety and strict regulatory compliance to foster broader industrial adoption and consumer acceptance. Furthermore, toxicity considerations necessitate strict dose control, and economic constraints associated with formulation and advanced delivery systems, such as encapsulation, add additional complexity to conducting in vivo studies.

The predominance of in vitro studies in the application of essential oils in food systems can be attributed to their cost-effectiveness, ease of experimentation, and high reproducibility. In vitro studies allow for controlled evaluation of antimicrobial activity without interference from complex food matrix interactions. In contrast, in vivo food system studies are limited, likely due to the binding and partitioning of EO compounds within proteins, lipids, and carbohydrates, which reduce bioavailability and antimicrobial efficacy. Additionally, sensory implications and regulatory constraints further restrict direct application in food matrices.

## 10. Current Challenges and Future Directions

Despite rapid progress in essential oils stabilization technologies, the practical application in food systems remains constrained by several scientific, technological, regulatory, and commercial barriers. Many studies demonstrate promising antioxidant or antimicrobial activity under controlled laboratory conditions. However, fewer validate performance in real food materials and post-harvest storage systems.

A main challenge highlighted by this review is the gap between the number of studies conducted under in vitro conditions and those validating the efficacy of EO-based delivery systems in real food matrices and postharvest storage settings. Consequently, the complex interactions between EOs, food components, and packaging materials remain poorly understood. So, a crucial question persists: *“How does the food matrix influence essential oil release kinetics, antimicrobial and antioxidant efficacy, and sensory quality?”* Therefore, the most critical and urgent priority for future research should be to bridge this translational gap by validating EO-based preservation strategies across various real food components (lipids, proteins, carbohydrates) and realistic storage conditions (relative humidity, temperature).

Furthermore, the natural variability of EO remains a major challenge, as it affects stability, biological activities, release pattern, matrix compatibility, sensory acceptability, safety, scalability, and regulatory compliance. In addition, the release kinetics are still underexplored. Indeed, release modeling is driven by mathematical models, while more advanced approaches, such as machine learning algorithms, provide reliable alternatives that can integrate realistic storage conditions, including temperature, relative humidity, food composition, and storage time. Therefore, in parallel with food matrix validation, future studies should go beyond reporting GC-MS alone to connect EO composition with release behavior, bioactivity, and food system applications. and more importantly, integrate sensory acceptability, dose control, and safety via rational designs using multivariate optimization approaches, including machine learning algorithms, to ensure consumer acceptance, regulatory compliance, and market readiness.

Lastly, the scalability readiness of EO-based preservation systems in food systems remains highly tamper-proof, with insufficient information on their commercial viability to promote their industrial adoption and commercialization. Therefore, long-term strategic research must be dedicated to resolving industrial scalability bottlenecks, moving from proof-of-concept formulations to standardized, validated, and commercially viable EO delivery systems by integrating rigorous techno-economic analyses alongside Life Cycle Assessment (LCA) to ensure both economic viability and environmental sustainability.

## 11. Conclusions

This review demonstrates that essential oil stabilization is a rapidly developing research field driven by the need to improve the stability, functionality, delivery systems, release control, and applicability of essential oils (EOs) in real food matrices. The bibliometric findings revealed a growing interest in emulsions/nanoemulsions, encapsulation, and active packaging (coatings and films), reflecting technological effort to address EO volatility, oxidation, and poor aqueous solubility. The study also highlights the emergence of non-contact or vapor-phase delivery systems to balance functionality, sensory quality, and applicability. This emergence is likely linked to their ability to mitigate phytotoxicity, improve sensory perception, and enhance adsorption capacity, thereby opening new avenues for the use of essential oils for food safety and quality. However, the current literature on EO-based systems in food systems remains strongly confined to in vitro assays (78%), missing opportunities for a deep, mechanistic understanding of how complex food macromolecules bind, adsorb, and partition EO-active components, thereby affecting biological activity. Consequently, industrial adoption is bottlenecked by a persistent sensory efficacy paradox, in which the EO doses required to control resistant pathogens often trigger organoleptic rejection or phytotoxicity. Furthermore, emerging high-area carriers (nanofibers, zeolites, metal–organic frameworks) remain at low technological readiness levels due to complexities in manufacturing scale-up, the lack of standardized kinetic release models, and the absence of food-safe regulatory frameworks. Therefore, future research should shift toward rational formulation design (dynamic predictions), prioritize vapor-phase or non-contact application systems, and promote industrial readiness through Techno-Economic Analysis (TEA) and Life Cycle Assessment (LCA) alongside real-world postharvest supply chain validation to ensure economic viability, environmental sustainability, and regulatory compliance.

## Figures and Tables

**Table 1:** Comparative overview of recent reviews (2021–2025) on essential oil stabilization and delivery strategies and published in Scopus and Web of Science.

Focus of Previous Reviews	Main Limitations	Contributions of the Present Review	References
Focuses mainly on Cinnamon essential oil delivery systems.	Limited to a single essential oil species.	Covers multiple essential oils, improving the generalization of information on EO applicability	[15]
Focuses on controlled-release delivery systems.	Limited to specific delivery systems such as nanoemulsions, encapsulation, or active packaging, with limited comparison across technologies	Compare diverse stabilization and delivery systems.	[16,17,18]
Focuses on plant-based strategies in meat and fish preservation.	Limited applicability across food systems	Focuses on food system applications	[19,20]
Based on one type of material.	Limited to specific biopolymer-based packaging materials.	Includes multiple biopolymers and synthetic polymers.	[21,22]
Focuses on biopolymers	Insufficient integration of carrier properties, EO chemistry, antimicrobial efficacy, and food matrix interactions.	Includes EO stability, functionality, and diverse stabilization strategies.	[23]
Based on regulatory standards for essential oils as food antimicrobials.	Limited real food system validation.	Link EO stabilization with food matrices, quality impact, safety, regulatory considerations, and practical application challenge	[24]

## Data Availability

All data used has been included in the article.

## Abbreviations

The following abbreviations are used in this manuscript:

ANNs	Artificial Neural Networks
GA	Genetic Algorithms
AI	Artificial Intelligence
SVMs	Support Vector Machines
ML	Machine Learning
CCRD	Central Composite Rotatable design
CCD	Central Composite design
BBD	Box–Behnken design
DSD	D-optimal design
EOs	Essential oils
PRISMA	Preferred Reporting Items for Systematic Reviews
MOFs	Metal–Organic Framework
UV	Ultraviolet radiation
ROS	Reactive oxygen species
O/W	Oil-in-water
W/O	Water-in-oil
HIPE	High internal phase emulsions
CEO	Cedarwood essential oil
